# An In-Silico Sequence-Structure-Function Analysis of the N-Terminal Lobe in CT Group Bacterial ADP-Ribosyltransferase Toxins

**DOI:** 10.3390/toxins11060365

**Published:** 2019-06-21

**Authors:** Miguel R. Lugo, A. Rod Merrill

**Affiliations:** Department of Molecular and Cellular Biology, University of Guelph, Guelph N1G 2W1, Canada; mlugoa@gmail.com

**Keywords:** mono-ADP-ribosylation toxins, C3-like toxins, C2-like toxins, ADP-ribosylation, CT-toxins, target substrate motifs, N-terminal α-lobe

## Abstract

The C3-like toxins are single-domain proteins that represent a minimal mono-ADP-ribosyl transferase (mART) enzyme with a simple model scaffold for the entire cholera toxin (CT)-group. These proteins possess a single (A-domain) that modifies Rho proteins. In contrast, C2-like toxins require a binding/translocation partner (B-component) for intoxication. These are A-only toxins that contain the E-x-E motif, modify G-actin, but are two-domains with a C-domain possessing enzymatic activity. The N-domain of the C2-like toxins is unstructured, and its function is currently unknown. A sequence-structure-function comparison was performed on the N-terminal region of the mART domain of the enzymatic component of the CT toxin group in the CATCH fold (3.90.210.10). Special consideration was given to the N-domain distal segment, the α-lobe (α_1_–α_4_), and its different roles in these toxin sub-groups. These results show that the role of the N-terminal α-lobe is to provide a suitable configuration (i) of the α_2_–α_3_ helices to feature the α3-motif that has a role in NAD^+^ substrate binding and possibly in the interaction with the protein target; (ii) the α_3_–α_4_ helices to provide the α_3/4_-loop with protein-protein interaction capability; and (iii) the α_1_-N_tail_ that features specialized motif(s) according to the toxin type (A-only or A-B toxins) exhibiting an effect on the catalytic activity via the ARTT-loop, with a role in the inter-domain stability, and with a function in the binding and/or translocation steps during the internalization process.

## 1. Introduction

Bacterial mono ADP-ribosyl transferase toxins (mART toxins) belong to a family of toxins that catalyzes the covalent transfer of an ADP-ribose moiety from NAD^+^ to a protein or DNA target in a host cell, changing target activity and impairing target cell function and survival [1]. This toxin family includes diverse members such as exotoxin A (ExoA) from *Pseudomonas aeruginosa*, pertussis toxin (PT) from *Bordetella pertussis*, and cholera toxin (CT) from *Vibrio cholerae*. Members of this family are steadily increasing and are classified as either CT-group (after “Cholera toxin”) or DT-group (after “Diphtheria toxin”). 

All bacterial mART toxins share a common structural fold composed of ~100 residues with low sequence homology formed by a core scaffold of two perpendicular β-sheets, flanked by variable helical sub-structures and exposed-loops that constitute a cleft for the binding of the NAD^+^ substrate. The SCOP2 database [2] assigns mART toxins (SCOP2: 56400) to the α + β class of proteins (SCOP2: 53931) and to the “unusual” ADP-ribosylation fold (SCOP2: 56398). This fold is shared with eukaryotic mART proteins, Ecto-ARTs (SCOP2: 82814), and with the C-terminal domain of poly (ADP-ribose) polymerases known as PARPs (SCOP2: 56398), among others ADP-ribosylating proteins (for a review see Fieldhouse, 2008 [3]).

The CT-group of mART toxins is a large group of A-only toxins (containing the catalytic component only, e.g., C3bot1, Vis, ExoS) [4,5,6], and binary A-B toxins (A: catalytic, B: cell binding/translocation components in distinct proteins, e.g., *iota*, and CT) [7,8] and A/B (A and B components in the same polypeptide, e.g., Certhrax [9,10,11] with low sequence identity but containing three conserved regions with the consensus (Y/F)R (Underline refers to conserved residues) (Region 1), (Y/F)xSTS (Region 2), and the catalytic (Q/E)xE (Region 3) motifs, with either NAD^+^-binding or catalytic function (for reviews see Fieldhouse, 2010 [12]). The CT-group of toxins is classically divided into ExoS-like, C2-like, C3-like, and CT/PT-like subgroups of toxins, based mainly on the catalytic motif (Region 3), subunit organization, and macromolecule targets.

Exoenzyme S (ExoS) (UP: Q51448) (UP: UniProtKB accession number) from *P. aeruginosa* is an A-only, four-domain, bi-functional toxin/effector with a C-terminal mART domain; it requires factor- activating ExoS (FAS) for activation [13]. Three additional ExoS-like toxins have been identified: ExoT toxin (UP: Q9I788) from *P. aeruginosa* [6], the single-domain VopT toxin (UP: Q87G19) from *V. parahaemolyticus* [14], and AexT toxin (UP: Q93Q17) from *Aeromonas* sp. [15]. ExoS-like toxins are secreted into target cells via the type III secretion system (T3SS), possess the ExE catalytic motif and target the RAS family of G-proteins (except for AexT which targets G-actin) (for a review see Barbieri, 2004 [6]). 

C2 is a mART toxin (UP: D4N871) from *Clostridium botulinum* [16] and is an A-B multi-unit exotoxin whereas its C2I mART subunit (A-component) requires the C2II receptor-binding subunit (B-component) to facilitate cell entry of the catalytic A-subunit. The A-component is a bi-domain protein with both halves structurally, but not functionally equivalent. The C-terminal domain harbors the mART activity, while the N-terminal domain interacts with the B-component. The C2- like subgroup includes members such as *iota* toxin (UP: Q46220, Ia subunit) from *Clostridium perfringens* type E [17], CST toxin (UP: O06497, SA subunit) from *Clostridium spiroforme* [18], VIP toxin (UP: G8C882, VIP2 subunit) from *Bacillus cereus* [19], CDT toxin (UP: Q9KH42, CDTa subunit) from *Clostridium difficile* [20]; and the newly discovered CPILE toxin (UP: X512D7, CPILE-a subunit) from *Clostridium perfringens* W5052 strain [21]. C2-like toxins contain the catalytic ExE motif, form an AB_7_ oligomer to gain cell access, and ADP-ribosylate G-actin (for reviews see Tsuge 2017 [22]). 

The C3 exotoxin (UP: P15879) from *C. botulinum* [23] (C3bot1) is a single-domain (A-only) mART toxin. Eight other members of C3-like toxins have been described so far: C3bot2 (UP: Q00901) from *C. botulinum* [24], C3lim (UP: Q46134) from *C. limosum* [25], C3cer (UP: Q8KNY0) from *B. cereus* [26], C3larvin (UP: W2E3J5) and Plx2A (UP:M9V3B7) from *Paenibacillus larvae* [27,28], and three isoforms C3stau1(UP: P24121), C3stau2 (UP: Q8GAX6), and C3stau3 (UP: Q8VVU2) produced by *Staphyococcus aureus* [29,30]. C3-like toxins harbor the catalytic QxE signature and modifies RhoA, B, and C, among others GTPases (for reviews see Vogelsgesang, 2007 [31] and Tsuge, 2017 [22]).

The CT-like subgroup (after cholera/pertussis toxins) is a more heterogenous group in terms of the unit organization, catalytic motif, molecular target, and modified residues. This group includes CT (UP: P01555, A-subunit) and Chelt toxins (UP: A2PU44, A-subunit) from *V. cholera* [12,32], PT toxin (UP: P04977, A-subunit) from *Bacillus. pertussis* [33], human LT (UP: P43530, A-subunit) from *Eshcerichia coli* [34], Scabin toxin (UP: C9Z6T8) from *S. scabies* [35], among others.

However, there are other mART toxins that do not clearly match with any of the previous classification schemes for the CT group. In these “ungrouped” toxins are the following: SpyA toxin (UP: Q1J858/ Q1JN57) from *Streptococcus pyogenes* [36], which is structurally similar to C3-like toxins (A-only) and with a secretion signal-sequence, but harbors the ExE motif and ADP-ribosylates vimentin among other substrates [37], and Plx2A toxin (UP: M9V3B7) from *P. larvae* [28]. These proteins are related to C3-like toxins (single-domain A-component with QxE catalytic motif that ADP-ribosylate Rho proteins) but also show features of C2-like toxins (require a binding/translocation partner, B-component) for intoxication. SpvB toxin (UP: P555220) from *Salmonella enterica* [38], VahC toxin (UP: Q49TP5) from *Aeromonas hydrophila* [39], and Photox toxin (UP: Q7N9B1) from *Photorhabdus luminescens* [40] possess the ExE motif, ADP-ribosylate G-actin, and are two-domains toxins (C-domain has mART activity). AexU toxin (UP: A0FKE5) from *A. hydrophila* [41] has a domain organization like AexT and is secreted into target cells by using the type III secretion system and harbors the catalytic QxE motif rather than the ExE motif characteristic of ExoS- like toxins. Certhrax toxin (UP: Q4MV79) from *B. cereus* strain G9241 [9] is a two-domain A/B toxin with its catalytically C-domain homologous to C3-like toxins (QxE motif), but its N-domain is homologous to the PA-binding domain of the anthrax lethal factor from *Bacillus anthracis*. Vis toxin (UP: A3UNN4) from *Vibrio splendidus* strain 12B01 [5] is a single-domain A-only toxin homologous to C3-toxins with an N-terminal secretion signal peptide. Vis harbors the ExE motif, although Vis does not covalently modify conventional C2-like nor ExoS-like substrates. Mav toxin (UP: AOQLI5) from *Mycobacterium avium* strain 104 [12] is a tetra-domain A/B toxin with the mART activity in the C-terminal domain that contains the ExE catalytic motif. EFV toxin (UP: Q838U8) from *Enterococcus faecalis* strain V8583 [12] is a bi-domain protein with the mART activity in the C-terminal domain and an ExE catalytic motif that targets actin (unpublished results). 

The CATH database [42] assigns the closely related codes 3.90.176.10 for C2-, C3-, and ExoS- like subgroups, and 3.90.210.10 for the CT/PT-like subgroup of CT-group of mART toxins. The enzymatic component of the CT-group (CATCH fold, 3.90.176.10) at the N-terminal region, i.e., upstream of the strand β_1_ of the β-core scaffold, is well structured with a similar fold, albeit not much attention has been given to this section. In the present work, we performed a sequence-structure-function comparison of the N-terminal region of CT-group mART toxins in the CATCH 3.90.176.10 category (non-CT/PT-like toxins), with special emphasis on the N-most distal segment (a putative helix-coil structure) and its different roles in these toxins.

## 2. Results and Discussion

### 2.1. The Overall Structure of the N-Terminal α-Lobe

**Figure 1:**
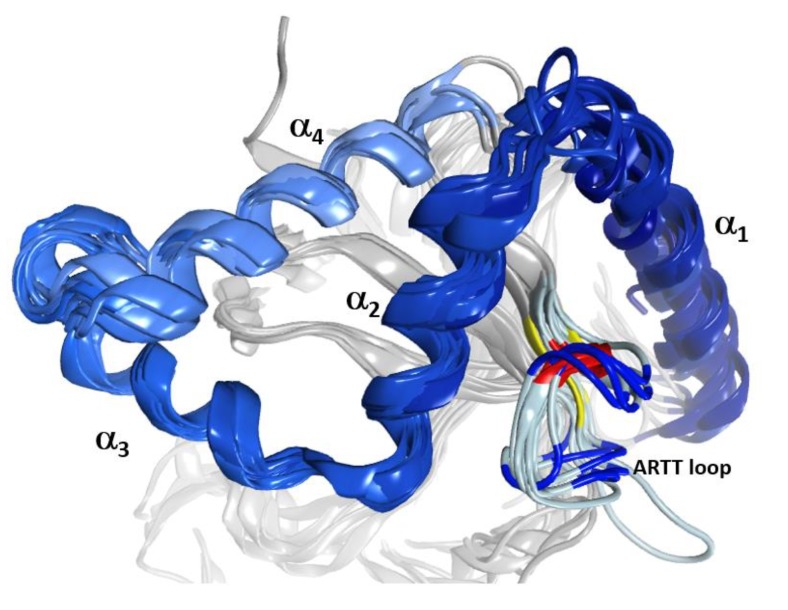
The α-lobe in C3-like toxins. Superposition of six C3-like toxins showing a compact N-terminal substructure that encompasses four α-helices. The ARTT loop (ADP-ribosyl-turn-turn) is a catalytic region that is responsible for the target substrate recognition and is shown at the bottom right of the overlaid structures. The superposed structures include C3 exotoxin (PDB:1G24) and C3bot2 (PDB:1R45) from *C. botulinum*, C3lim (PDB:3BW8) from *C. limosum*, C3cer (PDB:4XSG) from *B. cereus*, C3larvin (PDB:4TR5) from *P. larvae*, and C3stau2 (PDB:1OJQ) produced by *S. aureus*.

The C3-like toxins are single-domain proteins that represent a minimal mART enzyme with a simple scaffold that serves as the model for the entire CT-group. The N terminus is a helical region formed by four consecutive α-helices, α_1_–α_4_ (Figure 1). This region shares a lower average similarity for those residues in structured elements (~68%, from 52% to 88% pairwise) than with the set of β-strand residues that form the β-core superstructure (~73%, from 59% to 99% pairwise). Structurally, this N-terminal region consists of a conserved fold configured within a compact lobe, the “α-lobe”, with a high degree of overlap among the C3-like toxins (average RMSD_α_ = 2.4 Å; Figure 1).

**Figure 2:**
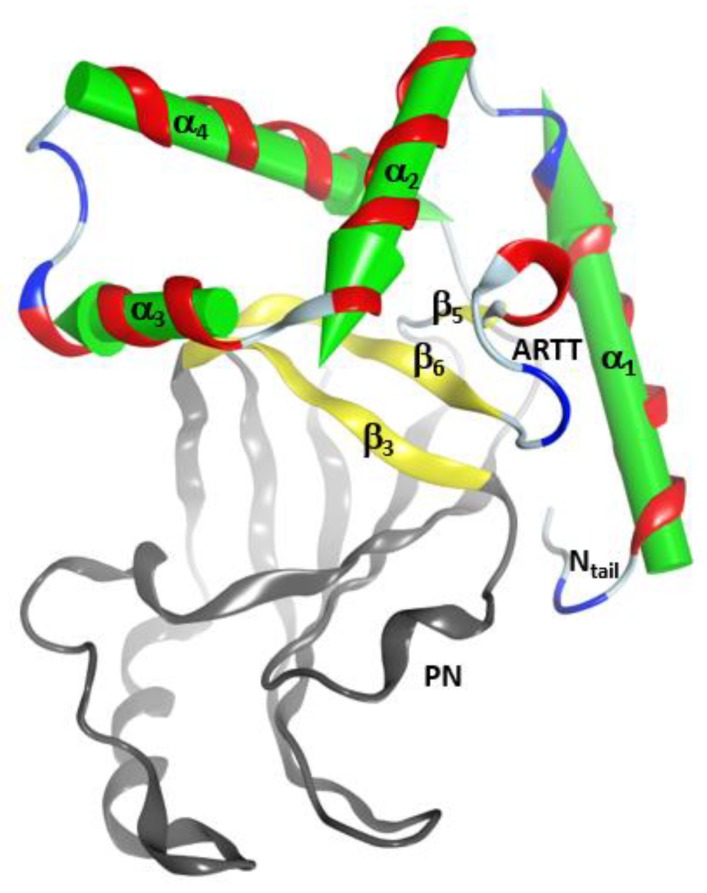
The α-lobe topology in C3-like toxins. Depiction of the V–L–αα-corner topology of the α- lobe. The green arrows correspond to the inertial axes of the α-helices. The βII sheet is shown in yellow ribbons with β3, β5, and β6 strongly interacting with the elements of the αα corner. The PN (phosphate-nicotinamide) loop is an NAD^+^ substrate-binding loop and it is indicated along with the N-terminus (N-terminal tail) of the domain.

This α-lobe is packed following a V−L−αα-corner topology (Figure 2), which encloses the β_II_ sheet and its connecting loops/turns according to: (i) an open V-shaped α_1_–α_2_ superstructure that surrounds the catalytic ARTT loop followed by (ii) an L-shaped α_2_–α_3_ superstructure that exposes the α_2/3_-turn and the key α3-motif (defined later) to the NAD^+^-binding pocket and to the protein target; and finally, (iii) an α_3_–α_4_-corner superstructure. The loop that links helices α_3_ and α_4_, the “α_3/4_-loop”, offers the flexibility required to form an αα-corner superstructure with a longer connection by turning the inertial axis of helix α_4_ orthogonal to both the α_3_ and the plane formed by the α_2_ and α_3_ axes. As a result, α_4_ is in a transverse orientation in relation to the β_II_ sheet, increasing the contact surface between the α-lobe and the β_II_ sheet. 

**Figure 3:**
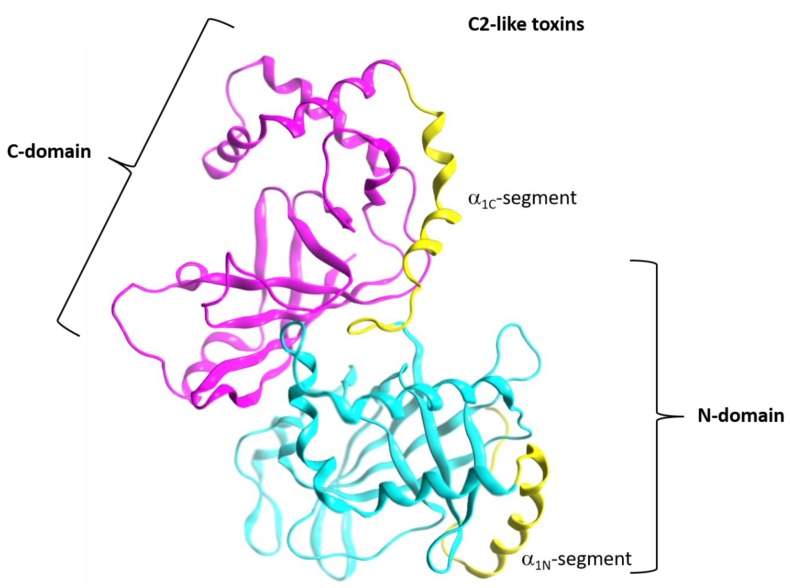
Topology of the A-component of the C2-like toxins. Structure of C2I toxin (PDB: 2J3Z) as a representative member of the C2-like class, showing the “adaptor” N domain (in cyan ribbons) and the “catalytic” C domain (in fuchsia ribbons). The T-segment of each domain is colored in yellow ribbons.

In addition, C3-like toxins are characterized by an unstructured N-terminal segment, the “N_tail_”, of variable length (Figure 2). Furthermore, the C3-like α-lobe is a well-structured, globular motif and is topologically identical or similar to: (i) the N-terminal helical region of the single-domain enzymatic component of the A-B binary Plx2 and Larvin toxins (Plx2A, PDB:5URP); (ii) the N- terminal helical region of the catalytic C-domain, “C2_C_-domain” of the A-component of the binary C2 toxin (Figure 3), C2I (PDB: 2J3Z), and C2-like toxins such as Vip2 (PDB: 1QS1), *iota* Ia (PDB: 1GIQ), CdtA (PDB: 2WN4), and SA component of the CST toxin (HM^2^). In these toxins, the N-terminal region of the non-catalytic N-domain, “C2_N_-domain”, interacts with the binding/translocation component (see later), shows a similar topology and superposes well with the C3-like α-lobes; it also possesses an additional well-defined helix (α_4a_) connecting α_3_ and α_4_; (iii) the helical region of the Certhrax toxin C-terminal domain (PDB: 4GF1)–Certhrax toxin has a significantly longer α_3/4_-loop than most C3- and C2-like toxins; and (iv) the helical region of the VahC C-terminal domain (PDB: 4FML) and SpvB (PDB: 2GWM). The first two crystallographically ‘solved’ helices in these toxins superpose well with the α_2_ and α_3_ helices of the α-lobe. There is also a 20-residue long helix-loop insertion between the second (equivalent to α_3_) and third (equivalent to α_4_) solved helices in these toxins. Additionally, the mART domain of Photox toxin reveals a high sequence homology with VahC and SpvB toxins; consequently, homology models of Photox show the canonical α_2_–α_4_ topology; and finally, (v) the N- terminal segment of Vis toxin (PDB: 4XZJ) which has a longer α_1_ helix with a slightly different orientation.

There is no X-ray structure for any member of the ExoS-like group. However, Sun et al. reported homology models of the N-terminal mART domains of ExoS and ExoT toxins, based on Ia toxin as a template; these models superpose well with the C3-like α-lobe [43]. On the other hand, for the enzymatic component of the CT/PT-subgroup (e.g., CT, LT-A/IIB, PT, and Scabin toxins, among others) there is no structural equivalent to the C3-like α-lobe. Instead, certain coils, helices, and strands (and their connecting loops) of the β_II_ sheet occupy the same location within the α-lobe without significant overlap with other elements—only the backbone structure of the α3-motif is roughly traced by an active-site helix or loop.

### 2.2. Stability of the α_2_−α_4_ Superstructure

**Figure 4:**
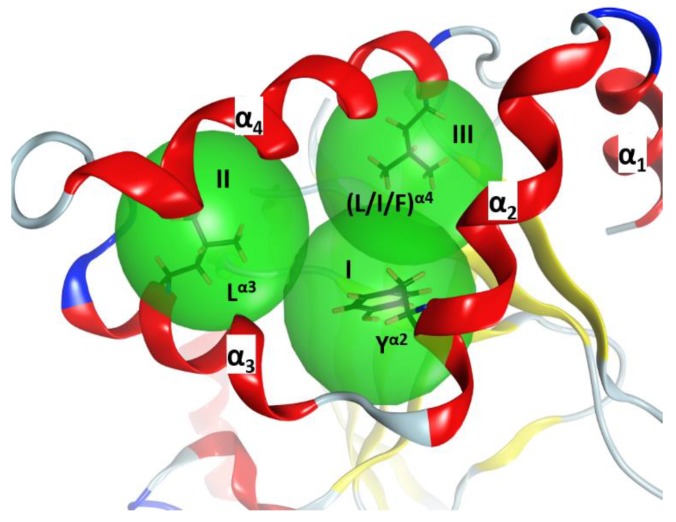
The α-lobe stability. α_2_–α_4_ superstructure of C3larvin (PDB: 4TR5), showing three hydrophobic clusters (depicted as green spheres) centered at three key residues. The structural “clusters” for the α_2_–α_4_ superstructure are designated as I, II, and III and the superstructure is framed by three α helices (α_1_, α_2_, and α_3_).

**Figure 5:**
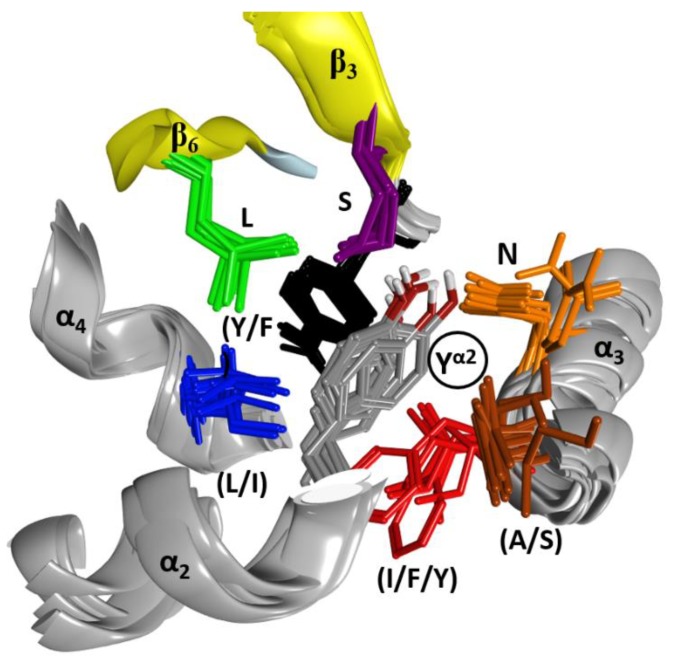
Interactions in Cluster_I_ of the α_2_–α_4_ superstructure. The cluster of residues around the conserved Tyr^α2^ (circled label; grey residue) is shown in this set of six overlaid C3 structures. The side- chains are shown for Tyr/Phe^β2,3^ (black), Ser^β3^ (mauve), Leu^β6^ (green), Leu/Ile^α4^ (blue), Ile/Phe/Tyr^α3^ (red), Ala/Ser^α3^ (brown), and Asn^α3^ (orange).

In C3-like toxins, the α_2_−α_4_ superstructure of the α-lobe is clustered and is in contact with the rest of the protein by a network of hydrophobic interactions centered in three key residues: Tyr in α_2_ (Tyr^α2^), Leu in α_3_ (Leu^α3^), and Leu/Ile/Phe in α_4_—one in each helix (Figure 4). In addition, an aromatic residue is present in the β_2/3_-turn, (Tyr/Phe)^β2/3^ (not shown). Effectively, Tyr^α2^ is the center of a cluster of interactions, “cluster_I_”, that cements the whole α_2_−α_4_ segment with the rest of the structure (Figure 5). The structural relevance of this hydrophobic and polar cluster of residues is evidenced by the large number of conserved and similar residues (including an invariant Leu in β_6_, see Figure 5) and the significant overlap of their side-chains. The reduced mobility of these residues as reported by their crystallographic *B*-factors reveals the mutually imposed constraint within the compact structure of the α-lobe. Tyr^α2^ is conserved in Plx2A in the C2_C_-domains, in most of the C2_N_-domains, and even in most of the toxins of the CT-group with known α-lobe topology. Compatible with its structural role, Tyr^α2^ is conservatively replaced with a Phe in Vis toxin, and with Leu in C2I_N_ and Vip2_N_ domains. However, Tyr^α2^ might also play an “active-site” role as its side-chain hydroxyl bridges the conserved Ser^β3^ (part of the STS motif) with Asn^α3^ (part of the NLR or α3-motif, see later); these are two critical residues involved in NAD^+^ substrate binding. The substitution of Tyr^α2^ with His^α2^ in HopU1 toxin is still compatible with the suggested role, particularly considering that HopU1 has a longer α_2/3_-link that might assist in the binding of NAD^+^ [44].

**Figure 6:**
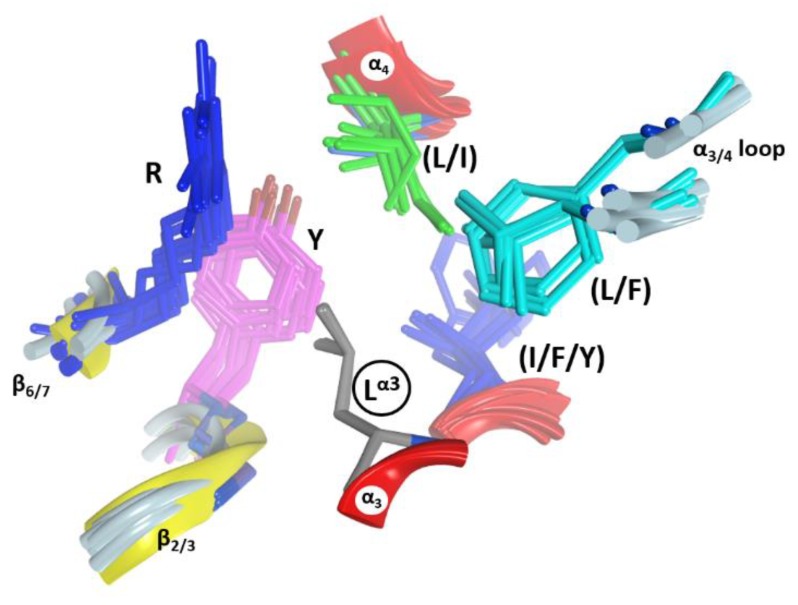
Interactions in Cluster_II_. The cluster of residues around the conserved Leu^α3^ (circled label) in Cluster_II_ is shown in this set of six overlaid C3 structures. The side-chains are shown for Arg ^β6/7^ (dark blue), Tyr ^β2/3^ (mauve), Leu^α3^ (grey), Ile/Phe/Tyr^α3^ (light blue), Leu/Phe^α3,4^ (cyan) and Ile/Val^α4^ (green). The residue nomenclature is as follows: the superscript refers to the alpha or beta secondary structure followed by the strand or helix number in the C3bot1 structure (PDB:1G24).

In C3-like toxins, the conserved Leu^α3^ is part of the (NLR)^α3^ motif and is the center of a second cluster of hydrophobic interactions, “cluster_II_” that links α_3_ and α_4_ and connects them to other elements (Figure 6). In this cluster, Leu^α3^ interacts with (Leu/Ile)^α4^, with some residues in the α_3/4_-loop, and with two other regions that act as hinges between both β-sheets–the conserved Tyr ^β2/3^ and the conserved Arg ^β6/7^ (part of the (LPR) ^β6/7^ motif). This cluster is structurally well defined in C3-like toxins as evidenced by superposing the structures on Leu^α3^ (grey residue in Figure 6). It is remarkable how identical this configuration (i.e., same side-chain torsion angles) is for these clustered residues. Leu^α3^ is conserved in Plx2A, the C2-like toxins (C2_C_-domains, but not the C2_N_-domains) and in most of the non-PT-like toxins (except, for example, the TccC3 and TccC5 toxins) [45]. This observation suggests an active role for Leu^α3^ in the stability of the α3-motif, which is relevant for the binding of the NAD^+^ substrate, rather than serving a pure structural role in the lobe stability.

A hydrophobic residue at the N terminus of α_4_, either Leu or Ile, is the center of the “cluster_III_” in C3-toxins and most non-PT-like toxins (not shown). The central (Leu/Ile)^α4^ contacts the conserved (Tyr/Phe)^β2/3^ (Figure 6) and the conserved Leu ^β6^ (Figure 5) in most toxins of the CT-group (an exception is HopU1 toxin) [46]. It is important to highlight (Tyr/Phe)^β2/3^ (Figure 6); this residue welds (coordinates) the three hydrophobic clusters in all the catalytic domains with the α-lobe configuration, even in the C2_N_-domains, which reaffirms the important structural role of this aromatic motif. Moreover, the presence of (Tyr/Phe)^β2/3^ as part of the (Y/F)xSTS motif in the toxins of the CT-group [3,12] reveals a structural role in preserving the N-terminal configuration, regardless of whether this region has the α-lobe topology in contact with the β-scaffold.

### 2.3. The α3-Helix and the α3-Motif

The α_3_-helix is defined in all the C2/C3-like toxins and most of the CT-like toxins with the α-lobe topology. In C2/C3-like toxins, the α_3_ helix harbors the α3-motif, Y^α2^−(IN−LR)^α3^ (Figure 5), which includes residues from both the α_2_ and α_3_ helices. The spatial orientation of the α3-motif along the α_3_ inertial axis offers a recognition surface aligned with the long axis of the bound pose of the NAD^+^ substrate; hence, functionally, the α3-motif binds the NAD^+^ substrate and contacts the target protein substrate. Thus, the α_2/3_-loop (N-terminal end), the conserved Asn^α3^ (at the center), and the semi- conserved Arg^α3^ (at the C-end) of the α3-motif, all point their side-chains towards the binding cavity and contact the bound NAD^+^ and/or the target protein substrate. In effect, in the complexes of C3bot1 (PDB: 2C8F) and C3stau2 (PDB: 1OJZ) with NAD^+^, the NH of Asn^α3^ H-bonds the NAD^+^ A- phosphate, and Arg^α3^ stacks with the NAD^+^ adenine ring. The relevance of these two residues in NAD^+^ binding is evidenced by their absence in the non-catalytic C_2N_-domains (see later). However, Asn^α3^ is absolutely conserved in all the non-PT-like toxins, while Arg^α3^ is less conserved (e.g., an Ile residue in certain C2-toxins). The role of Tyr^α2^ and of Leu^α3^ in this motif was already discussed above.

### 2.4. Role of the N_tail_-α_1_ Segment in C3- and C2-Like Toxins

Contrary to the conservation seen in the α2−α4 region, the N_tail_-α_1_ of C3-like toxins is a variable segment. The length of the unstructured N_tail_ is variable, being non-existent in C3larvin to 20 residues in C3stau(s) and C3cer. Likewise, α_1_ is 15 residues in C3lim, but is only eight residues in both C3cer (which superposes with the N-terminal half of C3lim α_1_) and C3larvin (which superposes with the C-terminal half of C3lim α_1_). 

The eight known C3-like toxins do not possess a specialized cell binding/translocation component or domain for access/entry into their target host cells, and the details on the mechanism and molecular determinants involved in the toxin internalization await further characterization (for a recent review see Rohrbeck, 2016 [47]). This is relevant for all C3-like toxins except C3stau toxins (C3stau1, C3stau2 and C3stau3), since *S. aureus* infects the host cell and releases the toxins into the cell cytoplasm [29]. Thus, C3bot1, C3bot2, C3lim, C3cer, and C3larvin toxins will be referred to collectively as “C3-etoxins” (with “e” after extracellular). 

**Figure 7:**
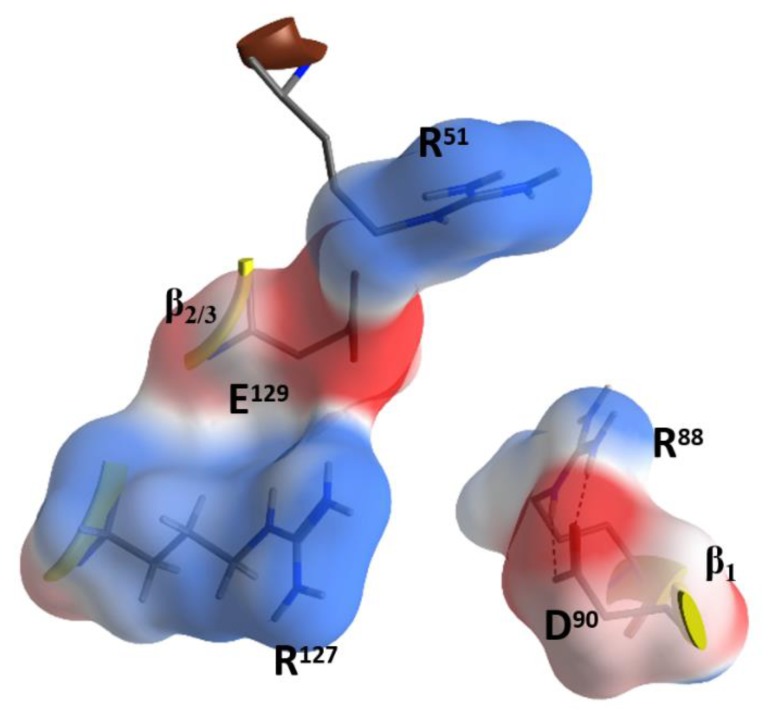
Putative vimentin binding motifs in C3bot1. Depiction of Arg88 and Asp90 of the RGD motif, the Arg127 and Glu129 of the RxE motif, and the conserved Arg51 in the signature α3 motif.

Due to the relatively high concentrations and long incubation times required for C3-etoxins to enter the target host cells, it was previously suggested that these toxins gain host access by non- specific pinocytosis [25]. Also, C3-etoxins have a basic α_1_ helix; basic peptides have been shown to interact with charged phospholipids on the outer layer of the cell membrane of host cells, causing destabilization of the lipid bilayer [48]. In fact, a short “transport” peptide fused to the C terminus of C3bot1 enabled chimeric toxin entry into the cytoplasm by a receptor-independent mechanism [49]. However, C3bot1 and C3lim may be selectively internalized into the cytoplasm of macrophage-like murine cells likely by a specific endocytotic mechanism [50]. Indeed, Rohrbeck and colleagues [51] identified a membrane partner that binds C3bot1, and vimentin (rod domain) was established as the cellular receptor in neuronal and macrophage cell lines. A more recent study showed the 88-RGD-90 sequence in C3bot1 functions as a vimentin binding-motif in neuronal cells [52]. Effectively the RGD motif is unique in C3-etoxins; however, it should be noted that this Arg residue (at β_1_) is an invariant residue in all CT-like toxins (the signature “R”) involved in the binding of the NAD^+^ substrate. The conserved Arg residue is buried in the NAD^+^-binding pocket and is stabilized by strong salt-bridges with the Asp in this motif in the apo structures. The fact that C3stau harbors RLL, instead of the RGD^β1^ motif, and only weakly intoxicates HT22 cells [53], would suggest that there is a concerted participation of the residues involved. In addition, because of the highly buried nature of the RGD^β1^ motif (e.g., Arg88 is only partially exposed to solvent), we envisage that other residue(s) that form the NAD^+^-binding interacting surface might participate in the recognition motif for vimentin. In this sense, the RGD^β1^ motif is close to the conserved R^α3^ of the signature α3-motif (Arg51 in C3bot1), and to the RxE motif located in the ^β^_2_ strand (residues 127–129 in C3bot1) of the C3 group, (Figure 7). The RGD^β1^ motif along with the RxE ^β2^ motif and R^α3^ form an ‘electrostatic clamp’ with complementary charges and H-bond capabilities in C3-etoxins (Figure 7). 

Interestingly, monomeric (soluble) vimentin is reported to be ADP-ribosylated by SpyA in the head domain of the protein [36,37]. This implies that vimentin must not bind into the NAD^+^-binding pocket of SpyA in order to behave as a protein substrate for the transfer reaction. Incidentally, SpyA lacks the RGD^β1^ motif (RYV^β1^ instead), lacks R^α3^ (D^α3^ instead), and lacks the RxE^β2^ motif (YxK^β2^). Additionally, no C2-like toxin exhibits these motifs without also binding vimentin. Plx2A harbors RGT^β1^, R^α3^ and LxE^β2^ and enters mouse macrophages in the absence of the Plx2B protein [28]. Therefore, the R^α3^ and E^β2^ residues may play a key role in the binding of the vimentin rod domain.

**Figure 8:**
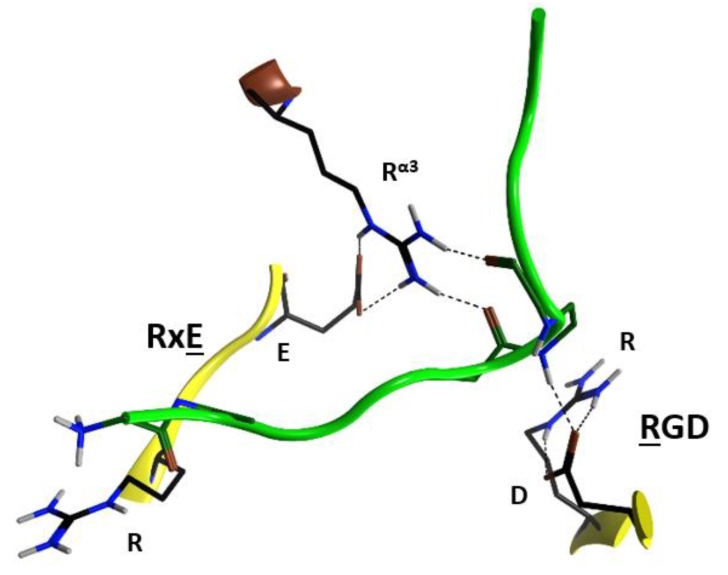
Protein–protein interactions in C3larvin. Depiction of the docking of a segment of C3larvin (green backbone) into the NAD^+^-binding pocket of another C3larvin molecule in the crystal structure (PDB: 4TR5). The residues correspond to the motifs presented in Figure 7.

Notably, the RxE motif (TxE in C3cer) is only present in the C3-etoxins. Thus, active-site residues, RGD (and likely also R^α3^ and RxE^β2^), while important for interaction with NAD^+^ for catalysis, have the role of binding vimentin on the membrane surface of the host cell. This dual function may be rationalized because these two activities are manifested in different compartments (i.e., the cytoplasm for enzymatic activity and the extracellular space for the vimentin recognition) and in different stages of the intoxication process. Therefore, considering that C3-like toxins correspond to a “minimal” toxin, then Asp90 and Arg127 (in C3bot1) may have evolved to use the invariant Arg51, Glu129, and Arg88 residues to bind a required membrane component for toxin internalization. Validation of the role of these residues in protein–protein interactions lies in the crystal contacts of a C3larvin fragment—of a symmetrically related molecule–docked into the NAD^+^-binding pocket in the C3larvin crystal structure (PDB: 4TR5) (Figure 8). 

There is additional evidence to suggest that the helical α-lobe might be part of the binding machinery with specific membrane component(s). In principle, the R^α3^ signature fulfills this proposal since R^α3^ is in the α-lobe. However, C3larvin fails to enter vimentin-expressing mouse macrophage cells [27] despite harboring RGD^β1^, R^α3^, and RTE^β2^. Notably, C3larvin possesses a truncated α_1_; a chimeric construct formed by adding 18 N-terminal residues (Ala1-Trp18) from the α_1_ helix of C3bot1 achieved cell penetration [27].

**Figure 9:**
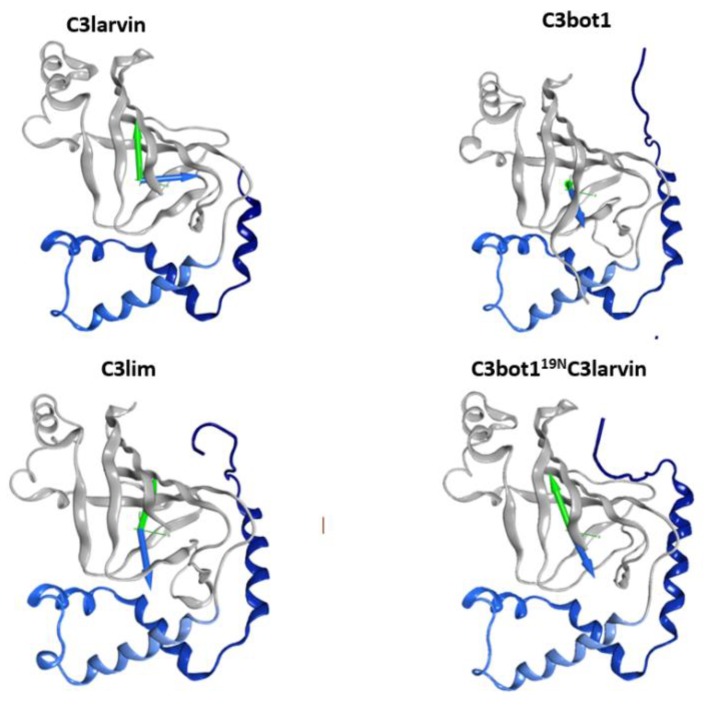
Dipole and hydrophobic moments for C3 toxins. The arrows represent the vectors of the electric (blue) and hydrophobic (green) dipoles moments for the wild-type (WT) and chimeric (bottom right) proteins.

Inspection of the short α_1_ in C3larvin reveals a negative residue at the N-terminus, Glu2, which is unique for this position (consensus Asn in C3-like toxins and Lys in C2-like toxins) and remarkably represents the only difference with the entire sequence of the Plx2A toxin, which shows excellent cell binding and penetration [28]. This Glu residue at one end and a cluster of basic residues at the other end of α_1_ confer a significant dipole-moment to the helix and suggest an electrostatic mode of interaction for this element. Notably, the calculated protein dipole moment for C3larvin points in a direction perpendicular to C3bot1 and C3lim dipole vectors (Figure 9); however, the protein dipole moment for the in silico C3bot1^18N^C3larvin chimera is practically aligned to the dipole vectors of C3bot1 and C3lim toxins. Thus, it seems that not only the net charge, but also the charge distribution may be important determinants for cell internalization.

The apparent role of the N_tail_-α_1_ in mediating toxin cell entry may not be exclusive to C3-etoxins. In the enzymatic component of binary C2-like toxins, the N- (adaptor) and C- (catalytic) domains face one another, so there is an obstruction in the central cleft (NAD^+^-binding pocket) of the N-domain, rendering it inactive (Figure 3). The N-domain of C2I toxin is considered a specialized structure that interacts with the binding/translocation protein partner (B-component) [54], and studies of chimeric fusion proteins with C2 toxin have revealed the α_N_-lobe of this toxin mediated cell entry. In effect, a construct formed by fusing the C2I_N_-domain with the C3lim toxin (C2I_N_C3lim construct) was able to intoxicate mammalian cells in the presence of the activated C2II protein (B-component) [55]. Moreover, the N-terminal α-lobe of the C2I_N_-domain (residues 1–87) alone was enough to facilitate uptake of the C2I_N_^87N^C3bot1 fusion protein [56]. Furthermore, a construct of C2I_N_ without the first 29 residues and the C3bot1 toxin, C2I_N_^Δ29N^C3bot1, failed to enter into enter HeLa cells [57]. Notably, the C3-like N_tail_-α_1_ element is preserved in the two domains of all C2-like toxins, and it is comparable in structure and relative location in each of them.

Plx2A toxin resembles C3-like toxins in the sense that its A-component is a single domain mART enzyme. Accordingly, the α-lobe of Plx2A is highly like the α-lobe of C3-like toxins, e.g., it possesses a small α_3/4_-loop (or α_4a_ helix), and the N_tail_-α_1_ is a terminal structure. However, at the same time, Plx2A resembles C2-like toxins because it requires a B-component, Plx2B, as a binding/translocation partner for the cell internalization [28,58]. In this sense, the advent of Plx2A offers an invaluable opportunity to assess the “properties” encoded in the terminal N_tail_-α_1_ segment of the C2- and C3-like toxins. 

**Figure 10:**
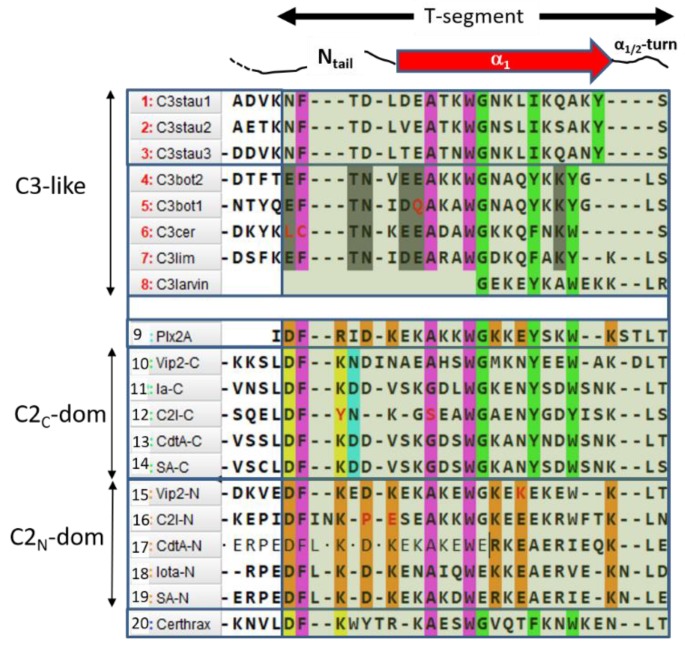
The T-segment motif. Multiple sequence alignment of the N_tail_-α_1_ segment of C3-like toxins (#1–#8), Plx2A toxin (#9), and C2-like toxins (#10–#19), and definition of the T-segment (translucent green box). The functional motifs are shown in color: purple, S-motif; green, C-motif; yellow and cyan, I-motif; light brown, B-motif; and gray, T-motif. Non-synonymous substitutions are highlighted in red text. Certhrax toxin (#20) is included for comparison.

**Figure 11:**
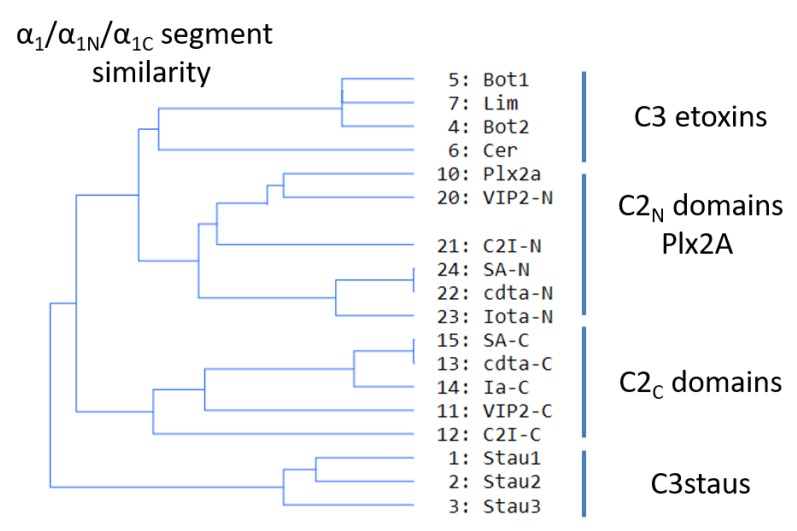
T-segment similarity. Cluster analysis of C3-like toxins (except C3larvin), C2-like toxins (N- and C-domains), and Plx2A, based on T-segment similarity.

Sequence-function analysis of the T-segment—a structure-based, multiple-sequence-alignment (MSA) enhanced with the pattern of molecular interactions—was performed for the N_tail_-α_1_ segment of the C3-like toxins, Plx2A, and the C2_N_- and C2_C_-domains (Figure 10). With the previous alignment and taking Plx2A toxin as the “master” sequence because it has the shortest N_tail_ among the aforementioned toxins, the “T-segment” (“T” after terminal) was identified that comprises five residues of the N_tail_, the α_1_ helix, and the α_1/2_-turn (Figure 10). Subsequently, a cluster analysis based on the similarity of the T-segment residues was performed on this toxin group, excluding C3larvin because it lacks the N_tail_ and has a truncated α_1_ (Figure 11).

Three noteworthy features of the T-segment include: (i) C3staus are unique from other domains/toxins; (ii) single-domain C3-etoxins cluster closer with C2_N_-domains than with the catalytic C2_C_-domains of C2-like toxins; and (iii) single-domain Plx2A toxin clusters with C2_N_-domains, rather than with the single-domain C3-etoxins or with the catalytic C2_C_-domains.

The T-segment in C3-etoxins can be defined by the consensus sequence (The long hyphen “–“ corresponds to any number of residues; like X_n_),

T_C3_-segment: EF^I^TN–(E/D)EA–W^II^G–(Y/F)^III^–KY^IV^

which comprises four (numbered as I to IV) highly conserved aromatic residues. Likewise, the T-segment of the N-domain of C2-like toxins can be defined by the

T_C2N_-segment: DF^I^–K–D–(A/G)–(K/R)–W^II^–K–E–K

and the T-segment of the C-domain of C2-like toxins can be defined by

T_C2C_-segment: DF^I^–K(N/D)D–A–W^II^G–Y^III^–(Y/W)^IV^−K

Thus, combining the three previous definitions, along with the T-segments of Plx2A, the following consensus sequences arise:

(S1) F^I^–A–W^II^G–(Y/F)^III^–(Y/W)^IV^, in Plx2A, and C3-etoxins,
(S2) DF^I^–D–(A/G)–W^II^G–Y^III^–W^IV^−K, in Plx2A, and C2_C_ domains, and
(S3) DF^I^–K–D–(K/R)–A–W^II^–(K/R)−E–K, in Plx2A, and C2_N_ domains.

**Figure 12:**
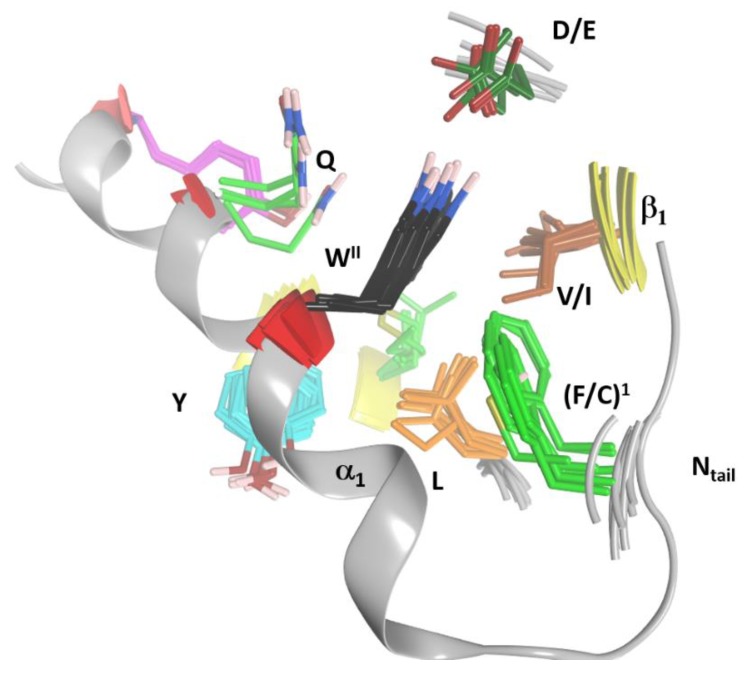
S-motif residues. Important residues that cluster around the two aromatic cornerstone residues of the S-motif. The cornerstone residues include Phe (mid-green) and Trp (black) and are surrounded by a cluster of residues, Gln (light green), Tyr (cyan), Leu (orange), Val/Ile (brown) and Asp/Glu (dark green).

**Figure 13:**
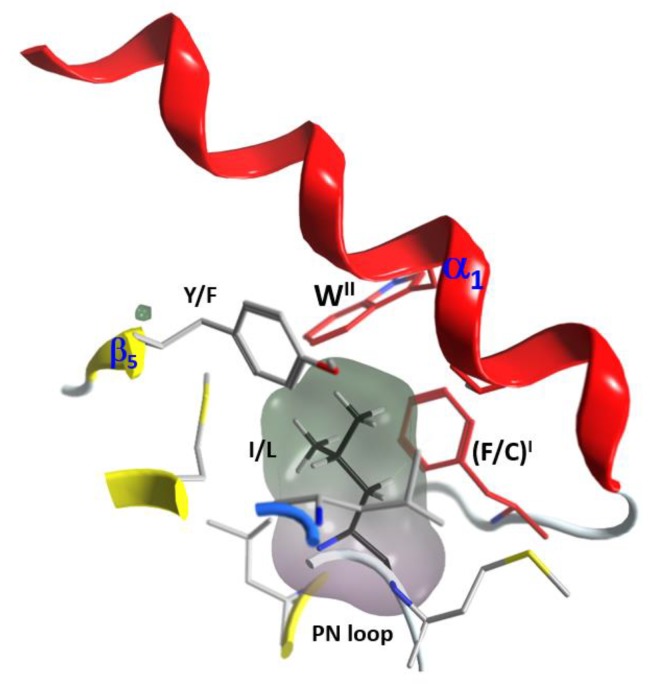
S-motif key interactions with PN- and ARTT-loops. Depiction of the interactions of the two aromatic residues (F^I^ and W^II^) of the S-motif with PN- and ARTT-loop residues (Y/F and I/L).

**Figure 14:**
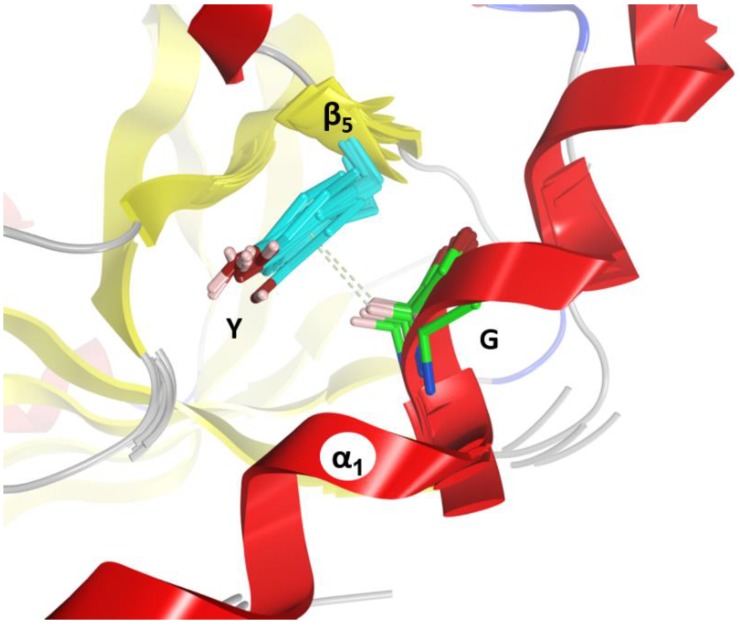
Conserved α_1_–ARTT interactions. Depiction of the H Pi-type H-bond between Gly (light green) of the C-motif and the conserved Tyr/Phe residue (cyan) in the ARTT-loop in C3-like toxins.

**Figure 15:**
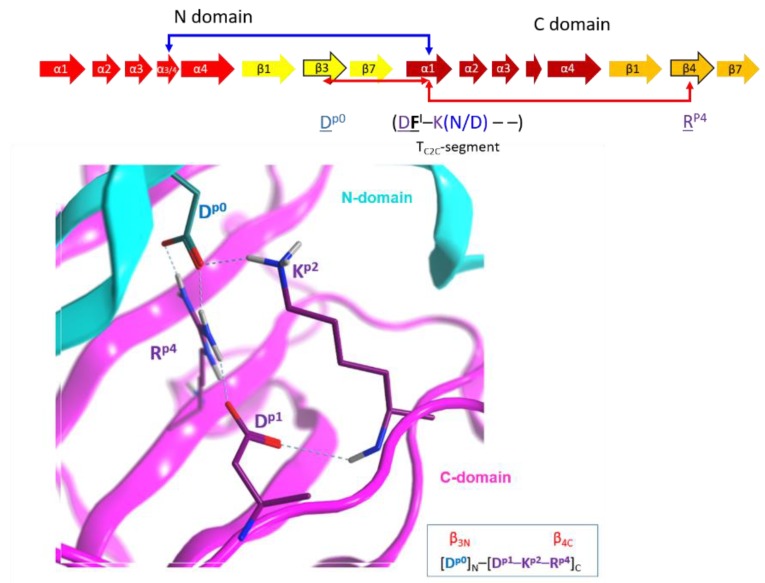
Inter-domain interactions. Depiction of the inter-domain interactions between the N-domain (D^p0^) and the C-domain (D^p1^K^P2^–R^p4^) residues of the I-motif that stabilize the structure of C2-like toxins.

**Figure 16:**
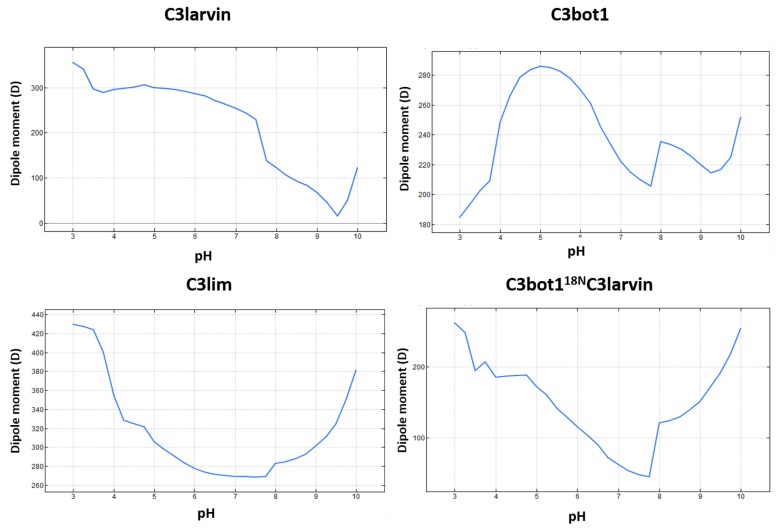
pH-dependence of the protein dipole-moment of C3 toxins. The pH profile of the electrical dipole-moment is shown for three C3-like toxins and for the chimeric protein consisting of the fusion of the N-terminus of C3bot1 with C3larvin toxin.

In turn, four sequence motifs emerge: 

(1) The “S-motif”, F^I^–(A/G)–W^II^, which is contained in the three S1−S3 consensus sequences motifs (fuchsia residues in Figure 10). The S-motif appears in all T-segments (including C3staus) and therefore likely participates in stabilizing the T-segment and neighboring regions (“S” after structure). In effect, Phe^I^ (Cys in C3cer) stabilizes the coiled N_tail_ segment with β_1_ in a β-like configuration. In turn, (Phe/Cys)^I^ along with Ala/Gly and the conserved Trp^II^ orient the α_1_ helix of the α-lobe by means of a network of hydrophobic and H-bond interactions (Figure 12). In particular, the interaction of the S-motif residues with both semi-conserved (Iso/Leu)^PN^ in the PN-loop, and with (Tyr/Phe) ^β5^ in the ARTT-loop (Figure 13) is relevant and will be discussed later. 

(2) The “C-motif”, G–(Y/F)^III^–(Y/W)^IV^ is found in the S1 and S2 consensus sequences and features residues not found in the S-motif (green residues in Figure 10). This motif only appears in catalytic domains/toxins, including C3stau’s (with Ile instead of (Y/F)^II^) and C3larvin, and therefore might be related to the stabilization of catalytic residues (“C” after catalytic). In effect, the conserved Gly residue fits into a small space and interacts/stabilizes the conserved Tyr^β5^ (Phe in C2I_C_) adjacent to the ARTT-loop by a H-Pi-type H-bond (Figure 14). The last two aromatic residues in the motif, (Tyr/Phe)^III^ and (Tyr/Trp)^IV^, in addition to stabilizing the α_1_–α_2_ superstructure, interact with conserved residues at or adjacent to the ARTT-loop (not shown). Thus, the C-motif might have evolved along with the ARTT-loop and any key determinant might have been lost in the co-evolution of the C2_N_-domains with the “atrophy” of catalytic signatures in the ARTT-loop.

(3) The “B-motif”, D–(K/R)–D–(K/R)–(K/R)−E−K, appears only in the S3 consensus sequence and includes residues not present in the S- or C- motifs (orange residues in Figure 10). This unique motif appears in the “terminal” T-segment of the binary toxins A-component—Plx2A and C2_N_-domains. Incidentally, no other basic or acidic residue located in any helix displays this degree of conservation in these toxins/domains (C2_N_-domains, Plx2A) but shows no consensus in the rest of toxins/domains (C3-like toxins and C2_C_-domains). The length/flexibility, exposure, electric charge, and H-bond capability of the B-motif residues qualify it as a candidate for the binding of the B-component and/or to mediate the translocation of the complex into the cytoplasm (“B” after binding). The B-motif is remarkable, since it confirms that Plx2A shares elements with binary C2_N_-domains that might be implicated in the host cell internalization process.

(4) The “I-motif”, D–K(N/D), has residues in the T_C2C_-segments that are not included in the previous motifs (yellow and cyan residues in Figure 10) and this motif participates in the inter-domain stabilization (“I” after interdomain). This motif can be defined by three positions (p_1_...p_3_) according to D^p1^–K^p2^(N/D)^p3^. The p_1_ and p_2_ positions of the I-motif might have evolved along with a residue in the β_3N_-strand (additional position p_0_) and a residue in the β_4C_-strand (additional position p_4_), to form a quaternary cluster, [D^p0^]_N_−[D^p1^−K^p2^−R^p4^]_C_ that stabilized both domains (Figure 15). In agreement with the previous assertion, Asp^p0^ is conserved in β_3N_ and does not appear in β_3C_; Arg^p4^ is invariant in β_4C_ and does not appear in β_4N_. Incidentally, Asp^p0^ and Arg^p4^ are absent in equivalent elements (strands β_3_ and β_4_, respectively) of single-domain toxins. In addition, the 3rd position of the I-motif, (N/D)^p3^, makes effective contacts with the α_3/4_-loop in the C2_N_-domains (α_3/4N_-loop) to stabilize both domains (see the next section).

(5) The “T-motif” E–TN–(E/D)E–K contains residues that are unique in the T_C3_-segments and are not found in the previous motifs (gray residues in Figure 10). The role of this motif might be related to the translocation (“T” after translocation), consistent with the previous section. Accordingly, the increased toxicity observed for C3bot1 and C3lim when the pH is reduced from 7.4 to 5.5 correlates with the increase in both the protein net charge and dipole moment (Figure 16). Furthermore, assessing the pH-dependence of the cellular toxicity, the calculated pKas for Asp90 and Glu129 (the two acidic residues in the proposed binding-motifs of C3bot1) are 2.5 and 2.8, respectively—these are too low to be responsible for the pH response. However, Glu8 and Asp13 (both in the T-motif of C3bot1) have calculated pKa values of 4.6 and 3.8, respectively. C3lim exhibits a higher pH- dependence than C3bot1 in intoxicating macrophage cells [59]. Incidentally, C3lim has four acidic residues in its T-segment, with pKa values that range from 3.7 to 4.0, while the acidic residues of the interacting motifs possess lower pKas of 2.6 and 1.4, respectively. Obviously, the alkaline nature of the T-segment is enhanced by the protonation (neutralization) of the acidic residues. 

### 2.5. The Putative Role of the α_3/4_-Loop

In the single-domain Plx2A, and C3-like toxins, the segment that connects helices α_3_ and α_4_, the “α_3/4_-loop”, is either short and unstructured (e.g., C3bot1) or contains a small 3_10_-helix configuring a loop-helix-loop motif (e.g., C3lim), called the LHL-motif that protrudes from the α-lobe [60]. In the C2_C_-domains, the α_3/4C_-loop is also short and unstructured (e.g., Vip2) or harbors a small α-helix (e.g., CdtA). In most of these toxins/domains, there is an abundance of Asn, Gly, and Pro residues, which imply a structural role in allowing the αα-corner that connects α_3_ with α_4_ (Gly and Pro allow the turns, while the uncharged, but polar Asn, is either exposed or buried). However, the presence of both charged and hydrophobic residues in the turn of the α_3/4_-loops of some C3-like toxins (e.g., C3bot1 and C3bot2), along with their solvent exposure, makes the α_3/4_-loop a probable interacting motif in these toxins. Indeed, even the short LHL-motif of C3bot1 mediates the non-enzymatic interaction of the toxin with the RalA GTPase [60]. 

**Figure 17:**
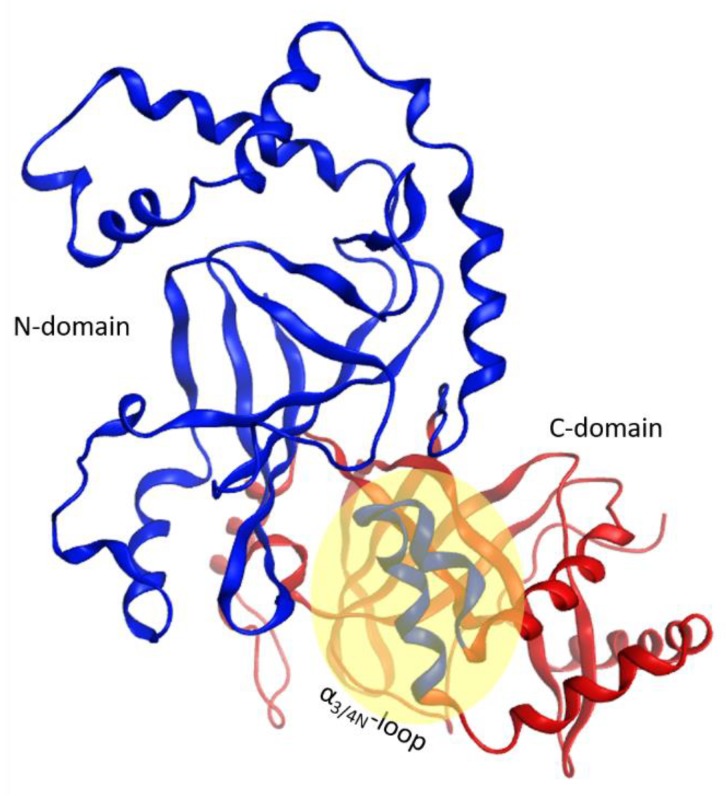
Inter-domain motif proximity. Identification of domains based on geometric criteria by the MOE 2018 algorithm, where the α_3_ and α_4a_ helices of the N-domain (in red ribbons) are part of the C-domain (in blue ribbons) in C2-like toxins. The yellow oval highlights the special proximity of the α_1_ of the C-domain with the α_3/4_-loop of the N-domain.

On the other hand, the α_3/4N_-loop is longer than its counterpart in C3-like toxins and C2_C_-domains and harbors an invariant Phe as well as other polar and hydrophobic residues. Interestingly, in Ia, CdtA, and SA toxins, the α_3/4N_-loop and α_4N_ are highly conserved with an average identity of 80.3% (90.2% similarity), and the Ia α_3N_−α_4N_ superstructure may be involved in the interaction with Ib (*iota* B-component) [61]. In effect, the segment responsible for the binding/internalization in Ib of *iota* toxin is not located at the N-terminus (T_C2N_-segment), likewise in C2I toxin (see previous section). Alternatively, in Ia toxin, this segment is more centrally located (residues 62–257), arising from α_3/4N_ (in the α_N_-lobe) to α_3C_ (in the α_C_-lobe) including both elements [61]. This postulate is reasonable if the α_3/4N_-loop can be considered part of the C_2C_-domain according to distance and packing criteria (Figure 17), such that the spatial proximity between helix α_1C_ and the α_3/4N_-loop may have a functional role. Notably, short segments defined by residues 42–177 (which includes α_3N_- and α_4N_-helices) and 222–257 (α_1C_−α_2C_ of the α_C_-lobe) may control binding to the Ib protein with a C3-like toxin chimera [61]. In addition, the (N/D)^p3^ residue of the I-motif interacts with the enlarged α_3/4N_-loop to stabilize the inter-domain architecture (see the previous section) with an invariant Ile residue in α_4aN_ (in α_4N_ for Vip2_N_).

### 2.6. The T-Segment in Other CT-Toxins

Certhrax toxin from *B. cereus* (PDB: 4GF1) is a bi-domain A-component toxin with the catalytically C-domain homologous to the C3-like toxin or the C2_C_-domain, but with the N-domain homologous to the PA-binding domain of anthrax lethal factor from *B. anthracis* [9]. Accordingly, Certhrax T-segment fulfills the S- and C-motifs of the canonical α_1_ configuration and catalytic ability (Figure 10). Incidentally, Certhrax T-segment lacks the T-(C3-like translocation) and B-(C2_N_-like binding/translocation) motifs; it exhibits inter-domain interactions via a modified I-motif. Thus, Certhrax clusters with the C2_C_-subgroup based on T-segment similarity (not shown), which is compatible with its catalytic non-terminal T-segment.

The X-ray structure of the mART domain of SpvB (PDB: 2GWM), a single-component actin-ADP-ribosylating toxin from *Salmonella spp*, lacks atomic coordinates of the region corresponding to the T-segment (i.e., upstream in the first “solved” helical structure, α_2_), and lacks the S- and C-motifs. Accordingly, an estimate of the secondary structure of this segment by the PSIPRED server predicted a coiled structure for this region, and several in-house homology models of the full-length SpvB from *S. typhimurium* report an extended coil for the T-segment.

Another mART actin-modifying toxin with known structure is VahC from *A. hydrophila* [39]. Unfortunately, the N-terminal truncated structures (PDBs: 4FML and 3NTS) lack the coordinates corresponding to the T-segment (i.e., upstream α_2_). The sequence of the VahC T-segment is highly like the corresponding segment in SpvB, and both segments harbor a poly-proline sequence. Likewise, Photox toxin from *P. luminescens* [40], has a T-segment that is highly similar and unstructured as found in both VahC and SpvB toxins. Interestingly, SpvB, VahC, and Photox lack the conserved Tyr^β5^, which is part of the canonical α_1_ configuration with catalytic ability (i.e., C3-like and C2_C_-like toxins, including Plx2A, and Certhrax). The phenol side chain of Tyr ^β5^ is the H-acceptor from the conserved Gly (backbone) in the α_1_-motif (Figure 14). Thus, the G α^1^−Tyr ^β5^ pair is involved in the mutual stability of α_1_ and the ARTT-loop. Notably, VahC, SpvB, and Photox do not show GH activity [7,39,40,43] which might be related to an unstructured T-segment that does not enclose the ARTT-loop (see later).

**Figure 18:**
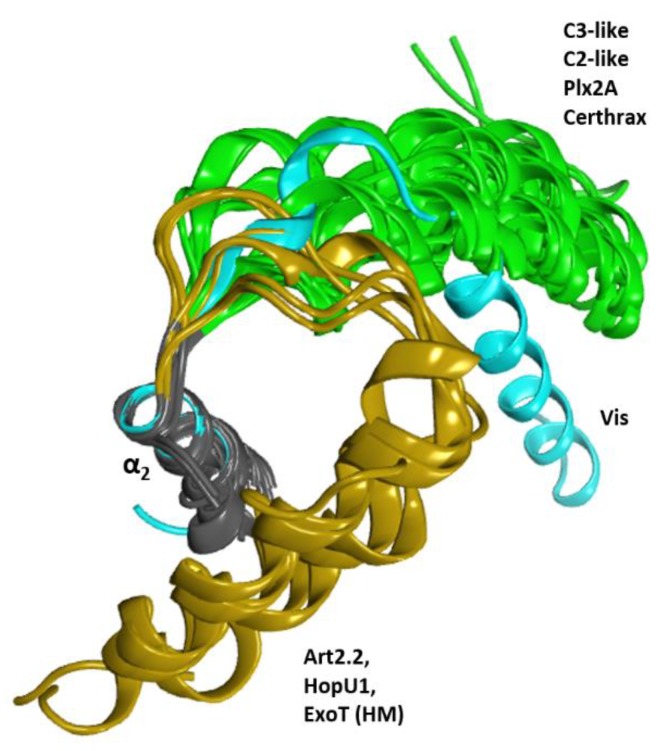
Conformations of the α_1_ helix. Superposition of several toxins of the CT-group for the α_2_ helix (gray ribbons) showing three conformation clusters for the α_1_ helix. These clusters are the canonical conformation in green, the altered conformation of Vis in cyan, and the folded conformation of HopU1, Art2.2, and ExoT (HM) in ochre.

Vis toxin from *V. splendidus* (PDB: 4XZJ) is a special case. The elongated α_1_ of Vis toxin follows a V-shaped configuration of the α_1_−α_2_ superstructure like C2/C3-toxins, although in a slightly ‘altered’ orientation (Figure 18). Accordingly, Vis has the aromatic W^II^ in its T-segment and does not fully qualify as an S-motif that would stabilize helix α_1_ in the canonical α-lobe configuration. Rather, the stabilization of α_1_ is achieved by an Arg residue in the N_tail_ that forms a salt-bridge with a Glu in the PN-loop and an Asp in the ARTT-loop (see later). Although it possesses some aromatic residues in the T-segment, Vis does not possess a C-motif and is unique in having the small, polar Ser residue replacing the conserved Gly. Notably, this Ser residue interacts in a similar manner with a residue (Thr^β5^, also a unique substitution in Vis) proximal to the ARTT-loop as observed for Gly in the C-motif.

In contrast, the HopU1 toxin from *Pseudomonas syringae* (PDB: 3UOJ) has α_1_ with an orientation diametrically opposed to the canonical conformation: an α_1_−α_2_ forming an αα-corner rather than the V-shaped configuration (Figure 18). The αα-corner configuration between α_1_ and α_2_ is facilitated by a long α_1,2_-loop (termed L1) [44] and is stabilized by a hydrophobic cluster of aromatic residues in the C-terminus of the T-segment (not shown). Although four aromatic residues are found in its T-segment, all the motifs in the canonical T-segment are absent in this toxin; notably, HopU1 lacks the S-motif that anchors α_1_ to the β_I_ strand and lacks the catalytic C-motif. Nevertheless, in HopU1 the N-end of the ARTT-loop (the end linked to strand β_5_) is stabilized by the αα-corner configuration, while the C-end of the ARTT-loop (the end linked to strand β_6_) is left partially exposed.

## 3. Conclusions

In summary, the role of the α-lobe is to provide a suitable configuration (location and orientation) of (i) the α_2_–α_3_ helices to feature the α3-motif that has a role in NAD^+^ substrate binding and possibly in the interaction with the protein target; (ii) the α_3_–α_4_ helices to provide the α_3/4_-loop with protein–protein interaction capability; and (iii) the α_1_-N_tail_, defined in the T-segment, that features specialized motif(s) according to the toxin type (A-only or A-B toxins) exhibiting an effect on the catalytic activity via the ARTT-loop, with a role in the inter-domain stability, and with a role in the binding and/or translocation steps during the internalization process. 

The canonical (C3-like) α-lobe configuration has the α_1_ helix forming a V-shape with α_2_ that surrounds the ARTT-loop. This configuration is stabilized by the ubiquitous S-motif in the T-segment of C3-like, C2-like (N- and C-domains), Plx2A and Certhrax toxin. Accordingly, other non-PT-like toxins (e.g., Vis and HopU1 toxins) with known structures show the canonical α_2_–α_4_ configuration. However, the α_1_ helix in an alternative configuration consistent with the lack of the S-motif in its T-segment. 

The presence of the catalytic signature on the ARTT-loop is not enough to guarantee GH- activity—the contrary is true—no catalytic residues in the ARTT-loop means no GH-activity. In non-PT-like toxins, GH-activity requires a stabilized ARTT-loop conformation, and this is achieved in toxins with the canonical α-lobe in the V-shape configuration of the α_1_–α_2_ helices, and specifically by the C-motif of the T-segments. In particular, the G^α1^−Tyr^β5^ pair is involved in the mutual stability of α_1_ and the ARTT-loop. Incidentally, C3larvin, which has a truncated α_1_, still has the C-motif and Tyr^β5^ and exhibits GH activity [27]; also, Certhrax has the canonical α_1_ configuration, Tyr^β5^, and C-motif and shows GH activity [9]. Accordingly, the alternative α_1_ conformation of Vis toxin encloses the ARTT-loop and shows GH activity [5]. On the contrary, VahC, SpvB, and Photox lack both the Tyr^β5^ and the C-motif (their T-segments are likely unstructured) and do not show GH activity [7,39,40,43]. HopU1 has an alternative α_1_ conformation and shows GH activity (unpublished data).

The specialization of the A-component of binary toxin classes (i.e., C2-like toxins) likely involves selective forces related to the intoxication mechanism that may dictate the composition of the T-segment. The bi-domain constitution might have arisen by gene duplication of an ancestral ADP-ribosyl transferase [62]; consequently, the T_C2N_- and T_C2C_-segments evolved to harbor residues appropriate for their location and roles. The T_C2N_-segment features the S- and B motifs. In effect, the inability of the C2_N_ domain to bind NAD^+^ must have forced the atrophy of the C-motif and the ARTT-loop (a required motif in the single-domain catalytic precursor), and instead, the T_C2N_-segment evolved to interact with the B-component. On the other hand, the T_C2C_-segment features the S-, C-, and I- motifs. In effect, the T_C2C_-segment might have evolved according to (a) a higher specialization of its ancestral kinetic role. Accordingly, the C-motif is better defined in the C2_C_-domains (G–Y^III^–W^IV^, with only the Y^IV^ variation in C2I_C_-domain) than in the single-domain toxins with the general definition; and (b) possesses an emerging structural role associated with the bi-domain topology—the I-motif. The I-motif, along with specific substitutions in the β_3N_ and β_4C_ strand, and with the α_3/4N_-loop, participates in inter-domain stabilization. 

It has been reported that the α-lobe of the C2I_N_ domain for C2 toxin and the RxG motif (possibly along with R^α3^ and the RxE motif) for C3bot1 may be the minimum sub-structure/motif necessary to stably bind to the membrane component—C2II and vimentin, respectively. In addition, the N-terminal T-segment may also participate in the process of internalization of both toxin groups into the host cell; it be involved in the translocation step from the early endosome to the cytoplasm. Accordingly, C3-like toxins feature the T-motif with acidic residues that might trigger the conformational changes required for membrane translocation; while C2-like toxins feature the B- motif. In this sense, the B-motif may bind to the B-component and/or to mediate the translocation into the cytoplasm. Intoxication experiments that monitor phenotypic alterations of the host cell do not distinguish which event is abolished when working with a toxin. Thus, in agreement with the translocation role of the B-motif, a construct of C2I_N_ without the first 29 residues and the C3bot1 toxin, C2I_N_^Δ29N^C3bot1, failed to be transported into HeLa cells, although binding of the construct to C2II on cell membranes was still observed [57]. Also, it is feasible that the α_3/4N_-loop is the sub-structure needed for binding (as has been observed in Ia toxin), while the B-motif is needed for the translocation step. 

## 4. Materials and Methods 

### 4.1. Ensemble of X-Ray Protein Structures

X-ray structures entries were downloaded from the Protein Data Bank (PDB). The datasets include high resolution (1.57 to 2.70 A) X-ray structures mainly of WT proteins in *apo* forms. However, in some cases proteins in different liganded states and diverse catalytically altered variants were also included for comparative purposes. When multiple molecules were presented in the asymmetric unit of some crystal forms, the most complete molecule was selected. 

### 4.2. Force-Field Settings and Structure Preparation

Protein preparation and molecular mechanics (MM) calculations were performed using the computational suite Molecular Operative Environment (MOE) release 2018.10 (Chemical Computing Group Inc, Montreal, CA, USA). The force field employed was the MOE Amber12: EHT, with AMBER12 parameters set (ff12) for protein, and parameters calculated from the Extended Hückey Theory for the NAD^+^ molecule and co-solvents. For the implicit solvent model, the Generalized Born-Volume Integral (GB/VI) formalism was employed, with dielectrics $\varepsilon_{\mathrm{pro}}=2$ for the interior of the protein.

When short sections of X-ray structures were missing in the PDB data files, the peptide segments were crafted by using built-in homology model procedures in MOE. Then, the full X-ray structures were protonated using the MOE Protonate3D module to assign the ionization states and tautomers of protein side-chains and to orient crystallographic water molecules (CWMs) at T = 300 K, pH 7.4 and 0.1 M of ionic strength, along with the GB-VI (Generalized Born-Volume Integral) solvation model and MMFF94 partial charges. The protonated structures were initially geometry-optimized by keeping backbone coordinates fixed, tethering all other heavy atoms with a 100 kcal/mol force constant (0.25 Å buffer), and then energy-minimized until an RMS gradient ≤0.001 kcal/mol/Å^2^. 

### 4.3. Others

The multiple sequence alignments (MSA) were also performed using MOE 2018 software and based on the overall matching of secondary and tertiary structural elements. For a higher resolution alignment of short segments, the procedure was enhanced from the pattern of common molecular interactions. All protein structures were rendered in MOE 2018 software.
